# Outcomes and Treatment Costs of Skilled Nursing Facility Patients with Pressure Injuries and Malnutrition

**DOI:** 10.1093/geroni/igab046.3629

**Published:** 2021-12-17

**Authors:** Kirk Kerr, Cory Brunton, Mary Beth Arensberg

**Affiliations:** 1 Abbott Nutrition, Columbus, Ohio, United States; 2 Abbott, Columbus, Ohio, United States

## Abstract

Skilled nursing facilities (SNF) provide care for individuals requiring skilled care while transitioning to a more permanent residence post hospitalization. This analysis shows that diagnosed malnutrition and pressure injuries (PI) adversely impact SNF patients’ health and recovery. Length of SNF stay, total charges, and discharge disposition were analyzed using SNF claims from 2016-2020 Centers for Medicare & Medicaid Services (CMS) Standard Analytical File databases. An average of 4.5% SNF patients had diagnosed PIs, and 4.9% had diagnosed malnutrition. Patients with diagnosed malnutrition were more likely to have PIs than patients without diagnosed malnutrition (11.9% vs 4.1%). Patients with PIs had higher charges ($12,304 vs. $10,937), were less likely to be discharged home (11.1% vs 18.9%), and more likely to be discharged to a hospital (15.8% vs 11.0%) or deceased (2.8% vs 1.6%). Patients with diagnosed malnutrition displayed a similar pattern for charges ($11,587 vs $10,969), and discharge to home (14.5% vs 18.8%), hospital (13.5 vs 11.1%) or deceased (2.8% vs 1.6%). Length of SNF stay did not differ between patients with and without PIs (18.5 vs 18.6) and was slightly shorter for patients with diagnosed malnutrition (17.3 vs 18.9). While higher probability of rehospitalization or death could impact these results, drivers behind these differences need further investigation. Because malnourished patients were more likely to have PIs and both PI and malnutrition are associated with poorer patient discharge outcomes and higher costs, efforts to identify malnutrition and implement proper nutrition interventions should be prioritized as part of SNF quality improvement initiatives.

